# Role of Us9 Phosphorylation in Axonal Sorting and Anterograde Transport of Pseudorabies Virus

**DOI:** 10.1371/journal.pone.0058776

**Published:** 2013-03-19

**Authors:** Radomir Kratchmarov, Matthew P. Taylor, Lynn W. Enquist

**Affiliations:** Department of Molecular Biology, Princeton University, Princeton, New Jersey, United States of America; Queen's University, Canada

## Abstract

Alphaherpes viruses, such as pseudorabies virus (PRV), undergo anterograde transport in neuronal axons to facilitate anterograde spread within hosts. Axonal sorting and anterograde transport of virions is dependent on the viral membrane protein Us9, which interacts with the host motor protein Kif1A to direct transport. Us9-Kif1A interactions are necessary but not sufficient for these processes, indicating that additional cofactors or post-translational modifications are needed. In this study, we characterized two conserved serine phosphorylation sites (S51 and S53) in the PRV Us9 protein that are necessary for anterograde spread *in vivo*. We assessed the subcellular localization of phospho-Us9 subspecies during infection of neurons and found that the phospho-form is detectable on the majority, but not all, of axonal vesicles containing Us9 protein. In biochemical assays, phospho-Us9 was enriched in lipid raft membrane microdomains, though Us9 phosphorylation did not require prior lipid raft association. During infections of chambered neuronal cultures, we observed only a modest reduction in anterograde spread capacity for diserine mutant Us9, and no defect for monoserine mutants. Conversely, mutation of the kinase recognition sequence residues adjacent to the phosphorylation sites completely abrogated anterograde spread. In live-cell imaging analyses, anterograde transport of virions was reduced during infection with a recombinant PRV strain expressing GFP-tagged diserine mutant Us9. Phosphorylation was not required for Us9-Kif1A interaction, suggesting that Us9-Kif1A binding is a distinct step from the activation and/or stabilization of the transport complex. Taken together, our findings indicate that, while not essential, Us9 phosphorylation enhances Us9-Kif1A-based transport of virions in axons to modulate the overall efficiency of long-distance anterograde spread of infection.

## Introduction

Alphaherpes viruses, including pseudorabies virus (PRV) and herpes simplex virus (HSV)-1 and -2, infect the nervous system and establish latent infections in ganglia of the peripheral nervous system (PNS) in their natural hosts. Spread of infection to new hosts requires reactivation of the latent infection and subsequent transport of progeny virions in axons to distant egress sites in the periphery [1]. Anterograde transport away from the neuronal soma in axons is essential for spread to innervated epithelial layers, or more rarely into the central nervous system (CNS) [2]. Given the polarized nature of mature neurons, i.e. distinct somatodendritic and axonal compartments [3], an active sorting mechanism has evolved to permit progeny virions to enter axons following replication. For PRV, anterograde spread of infection *in vivo* along chains of synaptically connected neurons is dependent on the small type II membrane protein Us9 [4]. The role of Us9 in axonal sorting and transport has been established *in vitro* at the cellular level, with studies reporting Us9-dependent axonal sorting and anterograde transport of viral glycoproteins, tegument, and virions [5], [6].

Enveloped viral particles transport within the lumen of vesicles, likely derived from the trans-Golgi network (TGN) [7]. Us9 incorporation into these transport vesicles is necessary for and directly promotes sorting into axons and anterograde transport [8]. Co-immunoprecipitation experiments have demonstrated a functional interaction between Us9 and the neuron-specific kinesin-3 motor protein Kif1-A [9]. In uninfected neurons, Kif1-A facilitates the anterograde transport of pre-synaptic and dense-core vesicles [10], [11] and is likely repurposed by Us9 during infection to modulate the axonal sorting and transport of virions.

The subcellular localization of Us9 is critical for protein functionality. Us9 is enriched in lipid raft membrane microdomains, localization to which is essential for Us9-mediated anterograde transport of virions [12]. In polarized neurons, Us9 is sorted into specific vesicular compartments of the Golgi and endosomal networks, and certain non-functional Us9 mutants have aberrant membrane localization patterns [9].

**Figure 1:**
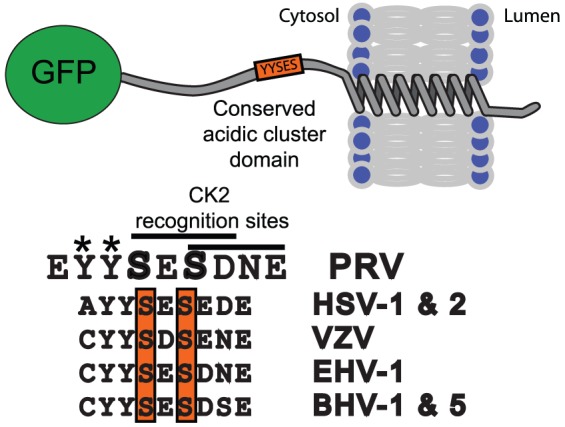
Schematic of the GFP-Us9 protein and critical residues. The topology and location of Green Fluorescent Protein amino-terminal fusion and the position of the critical and conserved acidic domain is presented. The majority of Us9 resides on the cytosolic side of the intracellular membrane, whereas a short carboxy terminal tail extends into the lumen of the vesicle. The acidic domain cluster resides just inside the transmembrane domain is highly conserved across alphaherpes virus species. For PRV Us9, the conserved tyrosines are critical for supporting anterograde transport and spread of virions. The serines located at positions 51 and 53 are highlighted, along with the conservation across alphaherpes viruses. The putative CK2 recognition sites delineated for each serine.

Though no crystal structure has been established for Us9, one critical domain is a 10-amino acid cluster of negatively charged acidic residues (46–55) [13] (Figure 1). Us9 mutants with the acidic cluster deleted fail to undergo anterograde spread *in vivo*. Two tyrosine (Y49 and Y50) residues within the cluster, known not to be phosphorylated or influence subcellular localization, are essential for productive anterograde spread *in vivo* [13] and are required for Us9-Kif1A binding [9] (Table 1).

**Table 1:** Compilation of PRV strains expressing mutant Us9 variants employed in this study as well as in previous work.

Strain	Description	*In Vitro* Spread	*In Vivo* Spread [13]
PRV Becker	Wild type	+++	+++
PRV 171	Us9 E52A, D54A, N55A, and E56A	−	+; delayed?
PRV 172	Us9 Y49,50A	−	−
PRV 173	Us9 S51,53A	+; delayed?	+; delayed?
PRV 340	Wild type GFP-Us9	+++	N/A
PRV 440	GFP-Us9 Y49,50A	−	N/A
PRV 451	GFP-Us9 S51,53A	+	N/A
PRV 452	GFP-Us9 S51A	+++	N/A
PRV 453	GFP-Us9 S53A	+++	N/A

Data on *in vitro* anterograde spread is from infection of compartmentalized neuronal cultures. All *in vivo* spread data from the rodent eye model system has been reported previously [13] and is presented here for comparison. +/− symbols denote phenotypes from robust spread (+++) to no spread (−).

Two serine residues (S51 and S53) within the acidic cluster are phosphorylated, as determined through radiolabelling assays [13], and are part of casein kinase-2 (CK2) consensus sequences. Phosphorylation of S51 and S53 is essential for anterograde spread *in vivo* [13] though it is unclear what role phosphorylation plays in the functional biochemistry of Us9 or Us9-directed anterograde transport. These serine residues may influence subcellular localization and/or facilitate interactions with binding partners. Furthermore, homologous, highly conserved serine residues exist in the Us9 protein of other related alphaherpes viruses, including HSV-1, -2 [14] and bovine herpes virus (BHV)-1, -5 [15] (Figure 1). It is also known that the varicella zoster virus Us9 homolog is phosphorylated by CK2 *in vitro* [16]. Three other minor serine phosphorylation sites previously undetected by radiolabelling (S38, S46, and S59) have also recently been detected in the PRV Us9 peptide through mass spectrometry [9]; however, these residues are not conserved with Us9 homologs from other alphaherpes viruses. Understanding the relevance of serine phosphorylation to Us9 mediated axonal sorting and transport will expand and refine our model of the molecular mechanisms that facilitate alphaherpes virus anterograde spread.

In this study, we characterized serine residues 51 and 53 of the PRV Us9 protein *in vitro* at the cellular and biochemical level. We visualized the distribution of phosphorylated Us9 in axons using immunofluorescence with a phospho-specific antibody and then analyzed the partitioning of phosphorylated and total Us9 protein species across different membrane microdomains. For live cell imaging and for quantification of anterograde spread, we isolated PRV recombinants expressing GFP-tagged, mutant Us9 variants with alanine substituted for one or both serine residues. We then performed infections of chambered neuronal cultures to quantify the spread defect associated with loss of phosphorylation. Finally, we assessed Us9-Kif1A interactions in the serine mutant background through co-immunoprecipitation experiments. This study of the role of Us9 phosphorylation in anterograde sorting, transport, and spread expands our understanding of the Us9 functional domains.

## Materials and Methods

### Plasmids and Viral Strains

We employed the wild type PRV Becker and derivative strains PRV 171 and PRV 173 expressing Us9 acidic cluster point mutants [13]. Several other mutant strains, which have been previously described, were also utilized, including: PRV 322, Us9 transferrin receptor transmembrane domain chimera [12], PRV 340, expressing GFP-Us9, as well as the dual fluorescent derivative PRV 341 (GFP-Us9/mRFP-Vp26) [8]. A defective mutant GFP-Us9 (Y49Y50-AA) (PRV 440) has also been described [8]. A recombinant adenovirus, AD TK101, expressing the same GFP-Us9 fusion protein, was used [9].

For this study, we constructed three new PRV strains (PRV 451, PRV 452, and PRV 453) expressing GFP-tagged mutant Us9 proteins. Site-directed mutagenesis of the GFP-Us9 N-terminal fusion plasmid pML122, originally used to construct PRV 340, was performed to introduce missense mutations (serine to alanine) at S51, S53, or both. Three sets of primers (forward and reverse) with the following nucleotide sequences were employed, (mutagenic codon underlined):

S51A:

F: 5′ GACTCGGACTGCTACTACGCCGAGAGCGACAACGAGACG 3′

R: 5′ CGTCTCGTTGTCGCTCTCGGCGTAGTAGCAGTCCGAGTC 3′

S53A:

F: 5′ GACTGCTACTACAGCGAGGCCGACAACGAGACGCCCAGC 3′

R: 5′ GCTGGGCGTCTCGTTGTCGGCCTCGCTGTAGTAGCAGTC 3′

S51,53A:

F: 5′ GACTCGGACTGCTACTACGCCGAGGCCGACAACGAGACG 3′

R: 5′ CGTCTCGTTGTCGGCCTCGGCGTAGTAGCAGTCCGAGTC 3′

Site-directed mutagenesis using the QuikChange II XL kit, performed according to the manufacturer's instructions (Stratagene, La Jolla, CA), yielded the plasmids pRK05 (GFP-Us9 S51,53A), pRK06 (GFP-Us9 S51A), and pRK07 (GFP-Us9 S53A). Each plasmid was sequenced to confirm mutagenesis and then recombined into the PRV genome through co-transfection with purified nucleocapsid DNA of PRV 337 [14], as previously described for inserting GFP-Us9 fusion proteins into the *Us4* genome locus [8]. Briefly, 293T cells grown to a density of ∼10^7^ cells per 10cm dish were transfected with PRV 337 nucleocapsid DNA and 4ug *NsiI*-digested pRK05, pRK06, or pRK07 respectively using Lipofectamine 2000, following the manufacturer's instructions (Invitrogen, Carlsbad, CA). After significant cytopathic effect (CPE) developed (∼48 hours post-transfection), the cells were harvested and the media plated onto PK15 cells at serial dilutions to isolate individual recombinant plaques expressing the GFP fusion protein. Final viral stocks were assessed by western blot (WB) for expression of the GFP-Us9 fusion and loss of BHV Us9 expression. Three strains were isolated: PRV 451 (GFP-Us9 S51,53A), PRV 452 (GFP-Us9 S51A), and PRV 453 (GFP-Us9 S53A).

Dual fluorescent strains expressing both serine mutant GFP-Us9 and the mRFP-Vp26 capsid fusion were constructed. PK15 cells were coinfected with PRV 451, PRV 452, or PRV 453 and PRV 325 (Us9-null, mRFP-Vp26, [8]). Cells were harvested after CPE and the media plated at serial dilutions on PK15 cells to visualize GFP/RFP dual-positive plaques. The resultant strains were designated PRV 454 (GFP-Us9 S51,53A/mRFP-Vp26), PRV 455 (GFP-Us9 S51A/mRFP-Vp26), and PRV 456 (GFP-Us9 S51,53A/mRFP-Vp26). A summary of the viral strains used in this study and their resulting phenotypes is displayed in Table 1.

### Antibodies and Reagents

Antibodies used in this study for western blot (WB) include rabbit polyclonal anti-Us9 serum ([17], 1∶1000), mouse monoclonal anti-KIF1A clone 16 (BD Transduction Laboratories, San Jose, CA, 1∶2000), mouse monoclonal anti-GFP (Roche, Mannheim, Germany, 1∶20,000), mouse monoclonal anti-β-actin clone AC-74 (Sigma Aldrich, 1∶5000) and goat anti-mouse or anti-rabbit-IgG HRP-conjugated antibodies (KPL, Gaithersburg, MD, 1∶20,000).

A novel phospho-specific Us9 mouse monoclonal antibody was isolated by Genscript (Piscataway, NJ) for this study. Briefly, BALB/c mice were immunized with a KLH-conjugated peptide mimicking the Us9 acidic cluster sequence, which contains the two putative serine phosphorylation sites [13]. The amino acid sequence of the synthetic peptide employed was QDDSDCYY(pS)E(pS)DNET, with “pS” indicating a phosphorylated serine residue. Mice exhibiting optimal immune responses were selected and 6 candidate B cell hybridomas were produced. Of the antibody clones, “2D5E6” (IgG1, K subtype) was selected based on ELISA analysis of its ability to discriminate phosphorylated from unphosphorylated Us9 and subsequently used at 1∶1000 for WB or 1∶100 for IF. Further confirmation of the phospho-specificity of 2D5E6 was obtained through parallel WB analysis of two Us9 samples derived from PK15 infected whole cell lysates, one of which was pre-treated with cow intestinal phosphatase (CIP) to remove any phosphate groups. CIP-treated Us9 samples were not detected by 2D5E6, indicating strong fidelity in the antibody's epitope (Figure S1).

### PK15, PC12, and dissociated primary neuron cultures

Porcine kidney 15 (PK15) cells were maintained in DMEM supplemented with 10% FBS and 1% penicillin/streptomycin. The transformed neuronal cell line PC12 [18] has been used extensively to model primary neurons for infection with PRV and reproduces Us9-associated viral transport phenotypes [6], [19]. For PC12 cultures, dishes were first coated with collagen (type 1) and washed 3 times with tissue culture-grade water to facilitate adherence. Undifferentiated PC12 cells were then plated and maintained in 85% RPMI, 10% horse serum, 5% FBS. To terminally differentiate PC12 cultures, cells were split 1∶5 and cultured in RPMI supplemented with 1% horse serum and nerve growth factor (NGF, Invitrogen, Carlsbad, CA) at 100 ng/ml. Differentiation media was replaced every 3^rd^ day for 11 days before infection. At this time, fully differentiated cells have exited the cell cycle and have developed neurites which exhibit axonal polarity, i.e. stain positive for the phosphorylated neurofilament H and Tau markers [19].

Dissociated cultures of primary rat superior cervical ganglia (SCG) neurons were prepared as previously detailed [19]. Prior to plating, MatTek glass-bottom dishes (Ashland, MA) were coated with poly-L-ornithine and murine laminin. SCG ganglia dissected from day E15.5 to E16.5 pregnant Sprague-Dawley rats (Hilltop Labs Inc., Pennsylvania) were then plated and maintained in neuronal media consisting of Neurobasal Media (Invitrogen) supplemented with 1% penicillin/streptomycin/glutamine (Invitrogen), B27 (Invitrogen), and 50 ng/mL NGF. Cultures were allowed to differentiate for at least 14 days prior to infection.

### Chambered neuronal culture

Campenot chambered cultures of SCG neurons have been employed extensively in our laboratory to assay directional spread of PRV [20]. For chambered cultures, plastic tissue culture dishes were coated as above for dissociated cultures and air-dried. A series of parallel grooves were then etched across the surface and covered with 1% methylcellulose in DMEM. A CAMP320 3 chambered teflon ring (Tyler Research; Edmonton, Alberta, Canada) was then coated with vacuum grease on one side and placed on top of the tissue culture surface, oriented such that the grooves extended across all three compartments. SCG neurons were then plated in one compartment (S compartment) and maintained in neuronal media as for dissociated cultures. Following ∼17 days of culture, robust axonal extensions develop across the center (M compartment), into the far side (N compartment). A detector layer of PK15 cells (∼5×10^5^ cells in neuronal media supplemented with 1% FBS) was plated in the N compartment 24 hours before any infections to amplify virus spread into this compartment.

### Viral Infections

Infections of PK15, PC12, dissociated and chambered SCG cultures were performed as previously described [8], [9]. All infections were performed at a high multiplicity of infection (MOI = 10) by adding the requisite volume of inoculum to the culture for 1 hour, after which the inoculum was removed and fresh media added back. Different timepoints post-infection were selected for different analyses and are described below.

### Lipid raft flotation assay

Isolation of detergent-resistant membrane (DRM) fractions, or lipid rafts, was performed through an OptiPrep sucrose gradient (Sigma-Aldrich, St. Louis, MO) [21]. A protocol previously employed for PRV-infected PC12 and SK cells was employed [12], [22]. Fully differentiated PC12 cultures were infected at an MOI of 10 for 16 hours, scraped into a 50 mL conical tube, and washed twice with cold RPMI. Cells were then lysed with 1 mL of lysis buffer (1% TX-100 in TNE buffer (25 mM Tris HCl, 150 mM NaCl, 5 mM EDTA) supplemented with protease inhibitor cocktail (Sigma-Aldrich) and 5 mM iodoacetamide. The lysate was homogenized by passage through an 18-gauge needle 15 times and allowed to rock for 30 minutes at 4C. Samples were then homogenized again and centrifuged briefly to pellet extraneous debris before being mixed with 2 ml of cold 60% Optiprep density gradient medium. The lysate-gradient mixture was then transferred to a Beckman SW41 ultracentrifuge tube and overlaid with 5 ml of cold 30% Optiprep gradient medium in TNE and a further 4 ml layer of 5% Optiprep gradient media in TNE. Samples were centrifuged at 200,000×g for 20 hours at 4C after which fifteen 1 mL fractions were collected, starting at the top of the tube. Subsequent WB assays were performed as below.

### Co-immunoprecipitation of GFP-Us9 complexes

To isolate and assess Us9 binding partners, we employed a recently described method that utilizes high affinity anti-GFP antibodies as probes for co-immunoprecipitation of viral protein complexes [23], [24]. Differentiated PC12 cells were infected with PRV strains expressing GFP-tagged Us9 variants, and cells were then lysed under the same conditions described for lipid raft flotation to preserve Us9 complexes. The methodology described by Kramer and colleagues [9] facilitates efficient detection of Us9 protein-protein interactions in samples prepared from differentiated, PRV-infected PC12 cultures, and we employed this protocol without modification.

### Western Blot analyses

Infected cell samples to be analyzed by WB without lipid raft flotation or co-immunoprecipitation were lysed with RSB, 1% NP40 detergent. Lysis conditions for lipid raft float or co-immunoprecipitation samples have been described above. All samples were then heated to 65C for 10 minutes, mixed 1∶6 with 6X Laemmli sample buffer, run on 12% 1-D SDS-PAGE gels, and transferred to a PVDF membrane. Antibodies and dilution factors employed are described above. Primary antibody was diluted in 5% milk in tris-buffered saline, supplemented with 0.1% tween (TBS-T), and applied to the membrane for 1 hour at room temperature. Membranes were then washed 3 times with TBS-T, and secondary HRP-conjugated antibody was applied for 1 hour at room temperature. After 3 subsequent washes with TBS-T, signal was detected using the chemiluminescent kit according to the manufacturer's instructors (Thermo-Fischer Scientific). For experiments requiring quantitation of WB signals, each protein sample was subject to a two-fold dilution series and the chemiluminescent signals were detected at each dilution to cover the linear and non-linear range of detection for the primary antibody.

### Immunofluorescence

Phosphorylated Us9 subspecies were visualized in axons of PRV-infected dissociated SCG cultures through immunofluorescence (IF). Cultures were infected with PRV 340 and fixed at 8 hours post-infection with 4% paraformaldehyde. Samples were washed 3 times with PBS and then blocked with 3% bovine serum album (BSA) in PBS for at least 1 hour before permeablization with 0.5% saponin in 3% BSA/PBS for 30 minutes. Primary antibody (mouse monoclonal 2D5E6) was diluted in the same permeablization solution and applied for 1 hour, followed by 3 washes with PBS. Secondary antibody (goat anti-mouse-IgG conjugated with AlexaFluor 568) was diluted in permeablization solution and applied for 1 hour, followed by 3 washes with PBS. Samples were then visualized with a Nikon Ti-Eclipse epifluorescent inverted microscope. As a negative control to establish non-specific antibody binding and background fluorescence, cultures were infected with PRV 451 encoding Us9 not detectable by 2D5E6 and analyzed under the same IF conditions.

### Live cell Fluorescence Microscopy Imaging

Live cell imaging of the recombinant fluorescent strains PRV 341, PRV 454, PRV 455, and PRV 456 was performed in dissociated SCG cultures as previously described for the wild-type GFP-Us9 fusion protein [8]. All imaging was performed with a Nikon Ti-Eclipse epifluorescent inverted microscope designed for rapid, serial acquisition of multiple fluorescent channels under a heated cell culture chamber (Ibidi; Martinsried, Germany). Cultures were imaged between 6–16 hours post infection. Manual quantitation of mobile/stalled capsids in 10 replicate movies from 2 biological replicates, each 3 minutes in length, for PRV 341 and PRV 454 was performed. Statistical analyses of capsids quantified from movies were performed using the Prism software package (GraphPad Software, Inc).

## Results

### Subcellular Localization of Phosphorylated Us9

**Figure 2:**
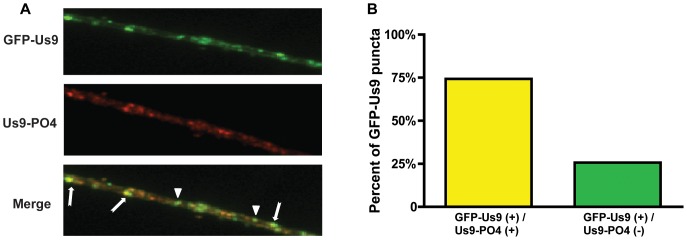
Subcellular localization of GFP-Us9 and phospho-GFP-Us9 in axons. A.) Representative IF visualization of total GFP-Us9 in axonal vesicles and phosphorylated Us9 subspecies detected by phospho-specific mouse monoclonal 2D5E6 at 8 hours post-infection with PRV 340. Discrete puncta are highlighted: arrows indicate dual positive GFP-Us9 (+)/Alexafluor 568 (+) structures, triangles denote single positive GFP-Us9 (+) structures. B.) Quantitation of phosphorylation status of Us9 puncta in axons. A total of 626 GFP-Us9 puncta were scored in axons and the percentage phosphorylated determined through IF. GFP-Us9 puncta were counted across 3 biological replicates.

We first utilized immunofluorescence microscopy to analyze the subcellular localization of phosphorylated Us9 subspecies (phospho-Us9) with the newly derived monoclonal phospho-specific antibody 2D5E6 (Figure 2A). At 8 hours post-infection of SCG neurons with PRV 340, vesicles containing either detectable or undetectable levels of phospho-Us9 were observed with no discernable difference in their distribution along the length of the axon. We quantified the proportion of phospho-Us9-positive vesicles as a percentage of the total GFP-Us9-positive vesicles and found that a majority (∼75%) but not all vesicles in axons contained detectable levels of phospho-Us9 (Figure 2B). The remaining structures could contain either unphosphorylated Us9, or low, undetectable amounts of the phosphorylated subspecies.

**Figure 3:**
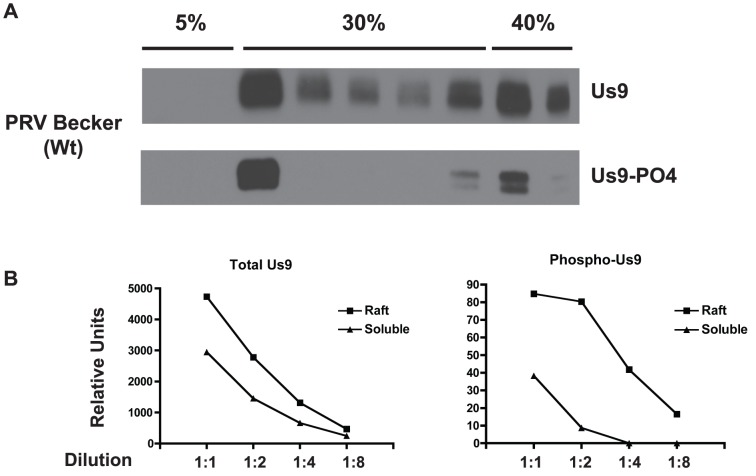
Distribution of Us9 and phospho-Us9 across membrane microdomains. A.) WB analysis following lipid raft flotation from differentiated PC12 cells at 14 hours post-infection with PRV Becker. Samples were collected from a discontinuous 5%–30%–40% Optiprep gradient. DRMs localize to the 5%–30% interface, while solubilized membrane proteins remain at the 30%–40% interface. Each 1 mL fraction from this gradient was run and probed with polyclonal anti-Us9 antibody to detect total Us9 protein content and phospho-specific monoclonal antibody to detect only phosphorylated Us9. B.) Quantitation of total Us9 and phospho-Us9 in insoluble raft membrane fraction and soluble fraction by WB for PC12 cells 14 hours post-infection with PRV Becker; values are reported as arbitrary chemiluminescence units from WB. Curves are representative of two independent lipid raft flotation experiments and show detection of each sample across a 2-fold dilution series. Dilution series covers detection of the respective Us9 protein from point of saturation, through the linear range of detection, to undetectable levels.

Previous studies have demonstrated that Us9 is enriched in lipid raft microdomains and that this localization pattern is essential for anterograde transport [12]. We therefore assessed the distribution of total Us9 protein and phospho-Us9 across membrane microdomains. Fully differentiated PC12 cultures were infected with PRV Becker and lipid raft isolations were performed at 14 hours post-infection. The Us9 content of the soluble and insoluble membrane fractions was then analyzed by quantitative WB. Total Us9 protein, detected by the rabbit polyclonal antibody, was present in the lipid raft and non-raft membrane fractions, as previously reported (Figure 3A). Phospho-Us9 was also detected in the lipid-raft and non-raft membrane fractions, though it appeared that more protein signal was present in the raft fraction (Figure 3A). However, a direct quantitative comparison could not be made given the difference in total Us9 protein content across these two fractions. Furthermore, direct comparisons between WB signals from the rabbit polyclonal and mouse monoclonal Us9 antibodies could not be made due to species and epitope differences. To circumvent these issues, we derived a relative ratio of phospho-Us9 signal in raft/non-raft membranes and normalized this to the relative ratio of total Us9 in raft/non-raft membranes (Figure 3B). The raft and non-raft fractions were each diluted 2-fold sequentially, and the dilutions were all probed with both antibodies by WB. When signal intensities were compared within the antibodies' linear range of detection, a two-fold enrichment of total Us9 was observed in the insoluble raft fraction as compared to the soluble fraction while four-fold more phospho-Us9 was detected in the insoluble raft fraction. After normalization for total Us9 protein content, we found a two-fold enrichment of phospho-Us9 in lipid rafts.

### Molecular mechanism of Us9 phosphorylation

**Figure 4:**
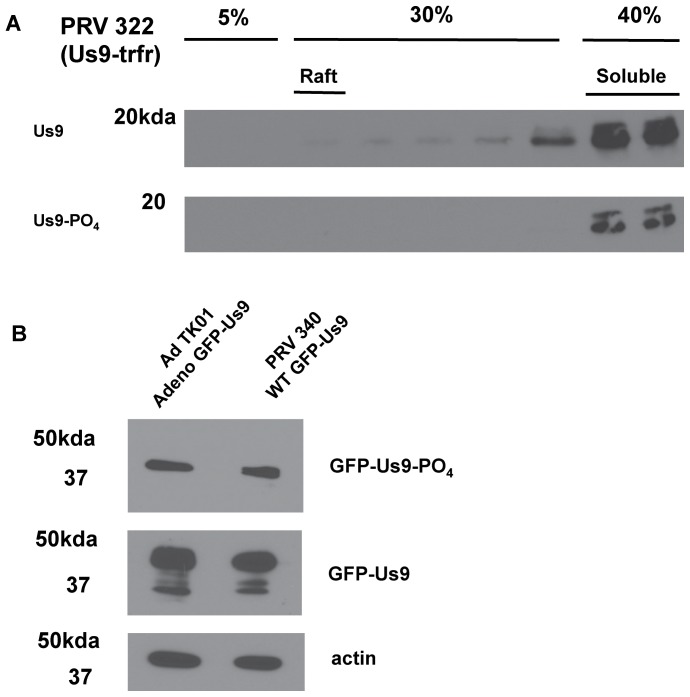
Molecular mechanisms of Us9 phosphorylation. WB analysis with indicated antibodies probing samples from: A.) Differentiated PC12 cells 14 hours post-infection with PRV 322 followed by lipid raft float preparation. Samples were collected from a discontinuous 5%–30%–40% Optiprep gradient. DRMs localize to the 5%–30% interface, while solubilized membrane proteins remain at the 30%–40% interface. Each 1 mL fraction from this gradient was run and probed with polyclonal anti-Us9 antibody to detect total Us9 protein content and phospho-specific monoclonal antibody to detect only phosphorylated Us9. B.) Whole cell lysates of differentiated PC12 cells transduced with Ad TK101 for 24 hours.

Phospho-Us9 is readily detectable in both raft and non-raft membranes, and it is unclear if this distribution reflects two different subpopulations or an equilibrium between association of the protein with different membrane microenvironments. To assess whether phosphorylation was dependent on association with a lipid raft, we infected differentiated PC12 cultures with PRV 322, expressing Us9 targeted to non-raft domains, and performed lipid raft isolation and WB analysis for phosphorylation as above. As previously described for PRV 322 infection [12], this Us9 mutant does not associate with lipid rafts (Figure 4A). Nevertheless, Us9 species targeted to non-raft membrane fractions were still phosphorylated, indicating that lipid raft association is not required for phosphorylation. We then confirmed that phosphorylation of Us9 is mediated by a cellular and not a viral kinase through WB analysis of differentiated PC12 cultures transduced with an adenovirus vector expressing GFP-Us9, as previously described (Figure 4B) [9].

### Anterograde Spread Capacity of Us9 Acidic Cluster Mutants

**Figure 5:**
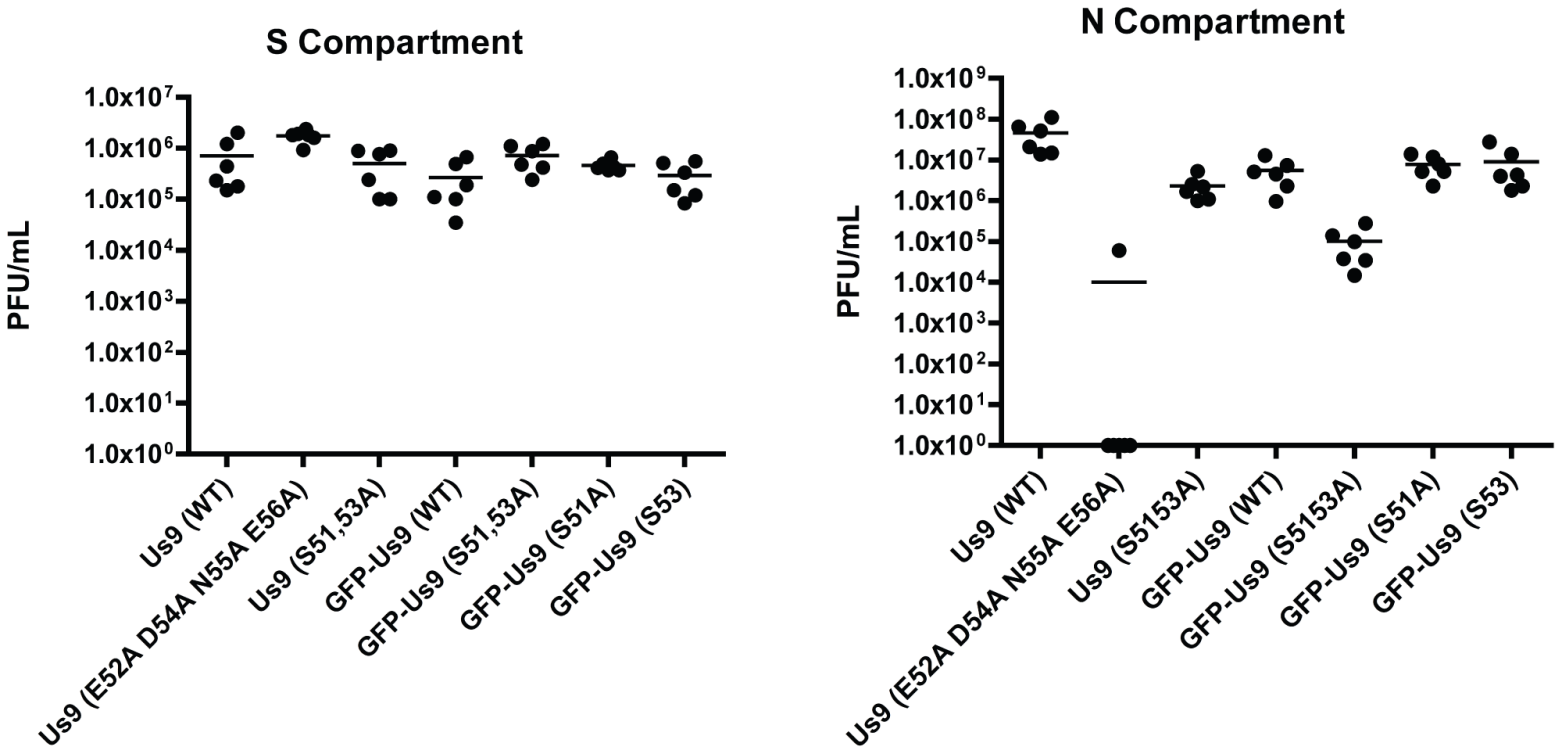
Anterograde spread capacity of Us9 acidic cluster point mutants. Viral titers in the S (left panel) and N (right panel) compartment of chambered SCG cultures 24 hours post-infection with: Untagged Us9: PRV Becker (wild-type), PRV 171 (Us9 E52A D54A N55A E56A), PRV 173 (Us9 S51,53A). GFP-tagged Us9: PRV 340 (GFP-Us9), PRV 451 (GFP-Us9 S51,53A), PRV 452 (GFP-Us9 S51A), and PRV 453 (GFP-Us9 S53A). Point estimates reflect viral titers in each compartment, representative of 2 biological replicates, each performed in triplicate. Line denotes median value.

To assess the functional relevance of phosphorylation to Us9-mediated anterograde transport of PRV, we infected the cell bodies of chambered SCG neuron cultures with a panel of recombinant strains expressing different Us9 variants. We assayed the anterograde spread capacity at 24 hours post-infection for PRV strains expressing wild type Us9 (PRV Becker or PRV 340) as well as several mutant Us9 variants (PRV 171, 173, 451, 452, and 453) (Figure 5). Anterograde spread of infection for PRV 173 was reduced by only 1.5log compared to wild-type PRV Becker, despite loss of the two serine phosphorylation sites. This finding clarifies previous *in vivo* work where PRV 173 was found to be defective for anterograde spread when compared to wild-type PRV Becker at equivalent timepoints but could undergo some spread at later times during infection [13]. PRV 171, expressing Us9 with several point mutations in non-phosphorylated acidic residues within the CK2 consensus site, exhibits markedly decreased anterograde spread capacity *in vivo* and is not efficiently phosphorylated [13]. Strikingly, we found a complete abrogation of anterograde spread for PRV 171 in chambered cultures, a phenotype that differs from that of PRV 173.

We then compared spread of infection for three GFP-tagged Us9 serine mutants to wild-type GFP-Us9. Loss of only one serine residue (51 or 53) had no detectable defect on anterograde spread (PRV 452 or 453). However, loss of both serine phosphorylation sites resulted in a ∼1.5log defect compared to wild-type GFP-Us9. Overall, we find that serine phosphorylation is not absolutely required for anterograde spread, though it may affect the kinetic efficiency of this process.

### Functional contribution of Us9 phosphorylation in anterograde transport

**Figure 6:**
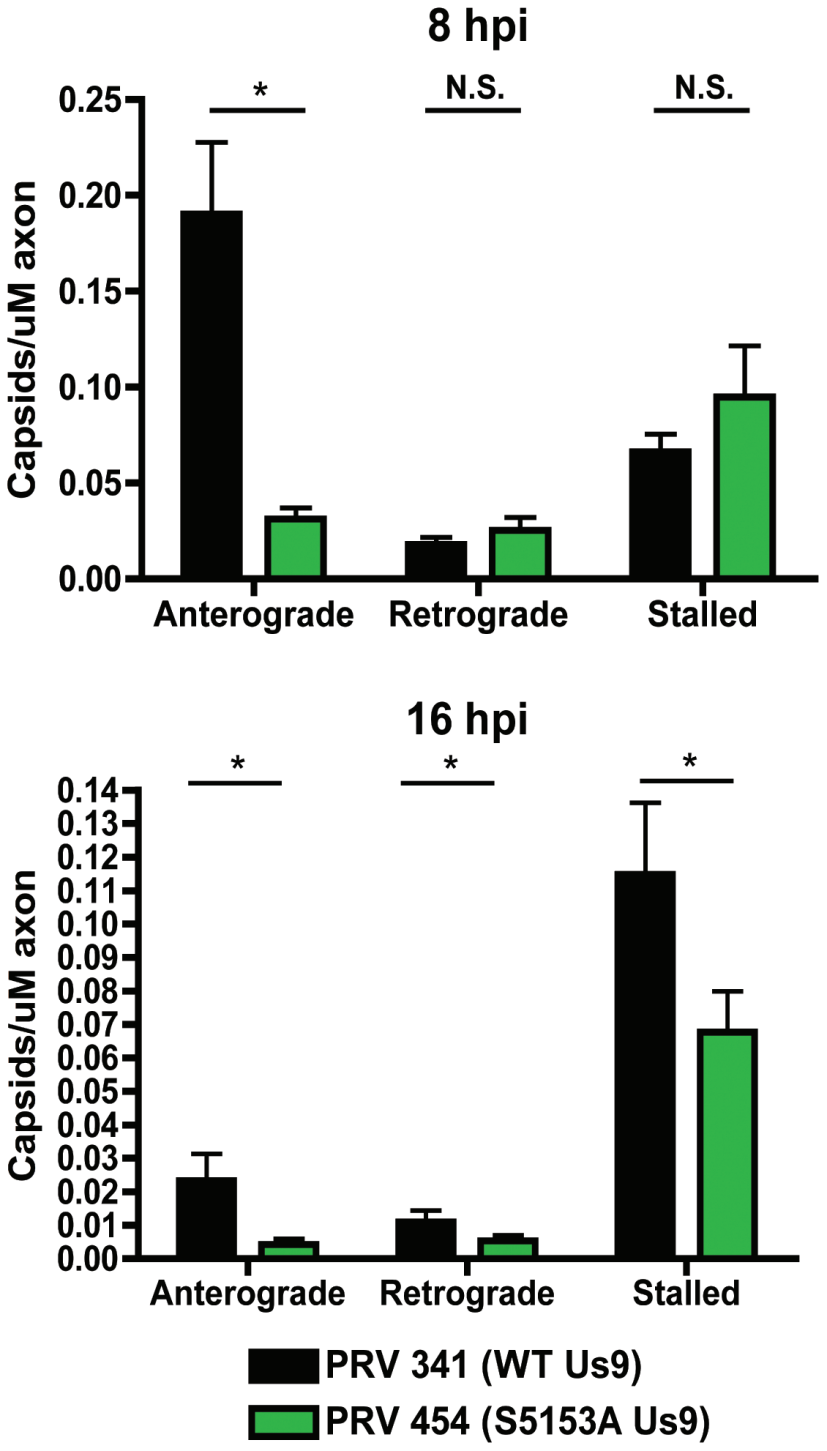
Quantitative live cell imaging of anterograde transport in diserine mutant Us9 background. Quantitation of anterograde transport of capsids in axons infected with PRV 341 (GFP-Us9/mRFP-Vp26) or PRV 454 (GFP-Us9 S51,53A/mRFP-Vp26) at 8 and 16 hours post-infection. 10 axons from two biological replicates were imaged for 3 minutes each at each time point and the number of anterograde, retrograde, and stalled capsids were manually quantified. Numbers of capsids were normalized to axon length in each movie. Error bars indicate +/− standard error of the mean (SEM). (*) indicates p<0.001.

To further characterize the importance of Us9 phosphorylation, we visualized virion transport during infection with several Us9 mutants. Dissociated SCG neurons were infected with PRV 341, 454, 455, or 456, and cells were imaged at 8 hours post-infection. No difference in the localization of Us9 was observed in cell bodies for these strains, with robust GFP-Us9 signal detected in the plasma membrane, as well as on intracellular vesicles (data not shown). Anterograde transport of mature virions, i.e. GFP-Us9 (+)/mRFP-Vp26 (+) punctate structures, was observed for PRV 454, 455, and 456 (Movies S1, S2, S3), although the number of transporting structures was substantially reduced for PRV 454. We quantified this transport defect by comparing the number of anterograde- and retrograde-directed capsids as well as stalled capsids observed during infection with PRV 341 or PRV 454 at 8 and 16 hours post-infection respectively (Figure 6). At the 8-hour timepoint, we observed no statistically significant difference in retrograde capsid transport between the two strains, and numbers of stalled capsids were also comparable. A significantly lower (∼4fold) amount of anterograde-directed puncta were observed at 8 hours post-infection with PRV 454, consistent with the reduced spread observed during infections of chambered neuronal cultures. At 16 hours post-infection, overall capsid puncta were reduced for PRV 341, but significantly more anterograde, retrograde, and stalled capsids were still detected as compared to PRV 454.

**Figure 7:**
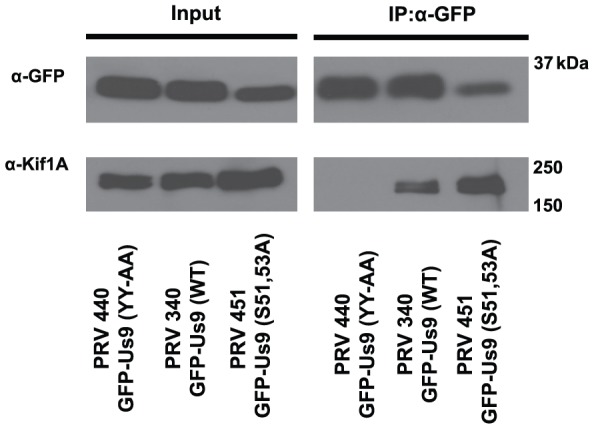
Phosphorylation of Us9 is not essential for Kif1A binding. Differentiated PC12 cells were infected with the indicated PRV strains, lysed at 12 hours post-infection, and subject to co-immunoprecipitation analysis using anti-GFP rabbit polyclonal antibodies. The ability of these mutant GFP-Us9 variants to interact with KIf1A was specifically assessed through WB detection.

As anterograde transport of virions was reduced during infection with diserine mutant Us9, we next determined if phosphorylation affects Us9-Kif1A binding, a key step in the axonal targeting of viral particles [9]. We used co-immunoprecipitation assays to assess interactions between diserine mutant Us9 and Kif1A. We infected differentiated PC12 cells with PRV 440, PRV 340, and PRV 451 and then assayed Kif1A-binding by the GFP-Us9 variants at 14 hours post-infection (Figure 7). As previously established, dityrosine mutant GFP-Us9 does not interact with Kif1A, while wild-type GFP-Us9 efficiently binds the motor. Kif1A was readily precipitated by the diserine mutant GFP-Us9, indicating that phosphorylation is not essential for Kif1A binding. However, given the observed deficit in transport associated with loss of serine phosphorylation, it is likely that the Us9-Kif1A transport machinery is not a simple mechanism but rather a more complex set of interactions and/or regulatory steps, which remain to be characterized. It is possible that phosphorylation acts downstream to facilitate activation of Kif1A and/or stabilization of the complex for more efficient transport.

## Discussion

### Role of Us9 Phosphorylation in Axonal Sorting and Transport

Understanding the molecular mechanisms underlying axonal sorting and subsequent anterograde transport of alphaherpes viruses is necessary for a rigorous characterization of viral spread and can complement *in vivo* studies of mutant viral strains. In this study, we characterized the role of Us9 phosphorylation at serines 51 and 53 in anterograde transport using biochemical fractionation and cellular imaging techniques that complement and expand upon the previously described spread deficiency attributed to these residues *in vivo* [13]. Taken together, our results indicate that phosphorylation of Us9 is not essential for its subcellular localization, binding to Kif1A, or anterograde spread. In isolation, neither serine 51 nor 53 appeared to be required for wild-type transport levels, but a 1.5 log defect in anterograde spread was observed after mutagenesis of both residues. This modest defect was also observed during live cell imaging of viral particle transport in the anterograde direction. Our findings suggest a model where phosphorylation of Us9 affects the efficiency of sorting and transport. Consistent with this idea, processive transport of host vesicles by mammalian Kif1A requires a release of autoinhibition in the protein dimer and may necessitate multiple copies of the motor protein per particle [25], [26]. As such, Us9-Kif1A interactions, measured by simplified co-immunoprecipitation assays, may not reflect more subtle components of the transport machinery such as motor activation status. While diserine mutant Us9 efficiently binds Kif1A, it may nevertheless be deficient for these other functional elements.

Interestingly, the Us9 phospho-serines differ from adjacent negatively charged residues in the defect associated with their mutagenesis. Here, we report that loss of the adjacent four negatively charged residues in the acidic cluster (PRV 171, (E52A, D54A, N55A, and E56A)) results in a complete abrogation of anterograde spread *in vitro*. Our previous model held that these sites formed the CK2 consensus sequence and were therefore necessary for phosphorylation of serines 51 and 53. However, since a single mutation of serine 51 or 53 has no effect on spread, and the dual mutant (S51,53A) has a modest defect, the acidic residues (E52, D54, N55, and E56) appear to have additional functions. We have reported that mutagenesis of the Us9 dityrosine motif (PRV 440) also results in a complete loss of Us9 function [8], likely mediated by a loss of Kif1A binding [9]. It is possible that the large, hydrophobic dityrosine motif may act in concert with the negatively charged residues to facilitate Kif1A binding. While sequence homology between Us9 and other proteins has been difficult to establish, the induced conformer transactivation domain of p53 shows some structural similarity to the Us9 acidic cluster in its arrangement of multiple negatively charged residues around two large hydrophobic moieties [27].

In this study we found that the diserine mutant Us9 has a modest anterograde spread defect compared to wild-type and efficiently binds Kif1A. However, *in vivo*, PRV expressing Us9 S51,53A does not spread to anterograde sites when compared to wild-type PRV Becker at equivalent early timepoints (Table 1). At later timepoints *in vivo*, serine mutant Us9 can facilitate some anterograde spread, suggesting that phosphorylation may affect the efficiency of transport. Our findings *in vitro* are consistent with such a model. Continued analyses of Us9 mutants could enable the molecular dissection of protein-protein interactions involved in virion transport, as modulated by the diserine, dityrosine, and acidic residue domains.

### Biochemical analyses of Us9 Phosphorylation

We found that Us9 is phosphorylated in cells when expressed by an adenovirus vector, though transduction of Us9 is not sufficient for Kif1A binding [9]. While we have shown that phospho-Us9 is enriched in lipid raft microdomains, raft localization is not required for Us9 phosphorylation. Us9 targeted to non-raft membranes by the transferrin receptor transmembrane domain is still efficiently phosphorylated. As the phosphorylated serine residues are located in the protein's cytoplasmic domain, it is unlikely that they directly affect association with rafts. Rather, phosphorylation may indirectly affect Us9's affinity for lipid rafts by augmenting its interactions with other binding partners, including itself. The degree to which Us9 proteins interact with each other is currently unknown, though the packing of multiple protein copies into lipid rafts might promote self-association. Further experimentation using fluorescence resonance energy transfer (FRET) could determine if Us9 self-associates, as well as the role of phosphorylation in governing Us9 protein-protein interactions.

### Anterograde Spread of Infection as Measured In Vivo and In Vitro

*In vivo* experiments using the rodent eye model of PRV infection revealed that Us9 was required for anterograde spread of infection and that certain domains of the protein were critical for functionality. The characterization of anterograde spread defects *in vitro* presented in this work expands our understanding of the molecular mechanisms underlying phenotypes observed *in vivo*. Both dityrosine and diserine mutant Us9 (PRV 172 and 173) were originally described as defective *in vivo* for anterograde spread compared to wild-type PRV at equivalent early time points [13]. However, at a later time point *in vivo*, diserine mutant Us9 was found to undergo some anterograde spread, suggesting a kinetic defect in efficiency of transport. Our work with live cell imaging and chambered neuronal cultures has clarified this observation and supports this conclusion. The convergence of *in vivo* and *in vitro* experimentation therefore indicates a more subtle function for the Us9 diserine domain, such as the activation and/or stabilization of interactions with binding partners.

## Supporting Information

Figure S1 **Detection of phosphorylated Us9 subspecies by the phospho-specific mouse monoclonal antibody clone 2D5E6.** WB analysis of PK15 whole cell lysates 12 hours post-infection with PRV Becker, with or without CIP treatment, using rabbit polyclonal Us9 antibody and mouse monoclonal phospho-specific Us9 clone 2D5E6.
(EPS)

Movie S1 **Live cell imaging of anterograde transport at 8 hours post-infection of dissociated SCG cultures.**
(MP4)

Movies S2 **Live cell imaging of anterograde transport at 8 hours post-infection of dissociated SCG cultures.**
(MP4)

Movie S3 **Live cell imaging of anterograde transport at 8 hours post-infection of dissociated SCG cultures.** GFP, RFP, and Merged channels are shown in each movie, played back at 5 frames per second using 300 ms exposures in each channel. Anterograde directionality is to the right in each movie. Movie S1: PRV 454 – GFP-Us9 S51,53A / mRFP-Vp26. Movie S2: PRV 455 – GFP-Us9 S51A / mRFP-Vp26. Movie S3: PRV 456 – GFP-Us9 S53A / mRFP-Vp26.
(MP4)
